# A Study on the Factors and Prediction Model of Triple-Negative Breast Cancer for Public Health Promotion

**DOI:** 10.3390/diagnostics13223486

**Published:** 2023-11-20

**Authors:** Young-Hee Nam

**Affiliations:** Department of Health Administration, Namseoul University, Cheonan 31020, Republic of Korea; yhnam14@nsu.ac.kr

**Keywords:** triple-negative breast cancer, hormone receptor, oncology, cancer registration, health promotion

## Abstract

This study was conducted to identify the risk causes and predictive models based on the clinical features of patients with breast cancer classified as triple-negative breast cancer (TNBC) and non-triple-negative breast cancer (non-TNBCs) using Korean cancer statistics. A total of 2045 cases that underwent three types of hormone receptor tests were obtained from Korean cancer data in 2016. Research data were analyzed with the software SPSS Ver. 26.0. TNBC and non-TNBCs accounted for 12.4% and 87.6% of the data, respectively. Tubular and lobular tumors occurred most frequently in the external quadrant of the breast (C50.4–C50.5; 43.1%). Compared to non-TNBCs, the incidence of TNBC was the most common in patients under the age of 39 (19.5%), followed by those over the age of 70 (17.3%). Tumors larger than 2 cm accounted for 16.0%, which was higher than the number of tumors smaller than 2 cm. Cases in stage IV cancer represented 21.7% of the data. Additionally, 21.0% of the patients were in the SEER stage of distant metastasis, which was the most prevalent SEER (surveillance, epidemiology, and end outcomes) stage. Neoadjuvant therapy was administered more frequently, with a rate of 24.1%. In the logistic regression and decision-making tree model, the variables that affected TNBC were age, differentiation grade, and neoadjuvant therapy. The predictive accuracies of logistic regression and decision-making tree models were 87.8% and 87.6%, respectively. In a decision-marking tree model, the differentiation grades of TNBC affect neoadjuvant therapy, and neoadjuvant therapy affects the cancer stage. Therefore, in order to promote the health of breast cancer patients, it is urgent to apply an intensive early health check-up program for those in their 40s and 50s with a high prevalence of TNBC. For patients with breast cancer, in TNBC cases, except for poorly differentiated cases, neoadjuvant therapy must be applied first at all differentiation grades. The establishment of a policy system is necessary for the success of this process.

## 1. Introduction

In 2021, the Korean Central Cancer Registry (KCCR) reported that approximately 36% of women would develop cancer during their lifetime. Breast cancer has the greatest incidence rate among female cancers, making up 20.6% of all malignancies [1]. It remarkably influences hormone receptors. Immunohistochemistry analysis reveals that cancerous breast tissues contain estrogen receptor (ER), progesterone receptor (PR), and human epidermal growth factor receptor-2 (HER2), which are receptors for female hormones. Thus, it is largely classified into breast cancer with female hormone receptors (ER, PR) and HER-2 and breast cancer without them, according to cancer that expresses these proteins [2,3,4]. On the basis of receptor status, hormone treatment, chemotherapy, and targeted treatment before and after surgery are customized [2,3,4].

Breast cancer that is positive for female hormone receptors (ER, PR) progresses more slowly than breast cancer that is not [4]. Cases positive for the ER and PR benefit significantly more from hormonal therapy than cases with only one positive aspect (ER+, PR− and ER−, PR+). If both are negative (ER−, PR−), the effect of hormone therapy cannot be expected [5]. When detected early, these cancers are often cured by anti-estrogen therapy and undergoing surgery or chemotherapy according to the stage [5]. However, since cancer cells may remain hidden in the bones, lungs, and liver for extended periods, patients must undergo long-term follow-ups to prevent recurrences. Premenopausal patients with large tumors, patients with rapidly growing cancer cells, and young patients are at high risk. Conversely, HER2-positive breast cancer, where the HER2 gene is amplified and/or overexpressed, is characterized by faster and more aggressive progression than other breast cancers. If targeted therapy is used in the early stages of cancer, the prognosis is good. It also lowers the risk of recurrence by more than 40% at stages I, II, and III [6].

As previously mentioned, TNBC does not express ER, PR, and HER2. It is the most hostile of all cancers; it has a reduced prognosis and various pathological features; it also progresses rapidly and often affects young patients [7]. Therefore, the treatment method for breast cancer should depend on the presence or absence of hormones. TNBC is difficult to treat because neither hormone therapy nor targeted anticancer drugs are effective. The chemotherapy process is employed to attack and destroy cancer cells using various strategies to carry out its cytotoxic effects. Chemotherapeutic drugs mostly stop cancer cells from proliferating quickly by interfering with vital biological functions, including DNA replication and mitosis. Furthermore, these substances cause cancer cells to undergo programmed cell death or apoptosis. Chemotherapy targets fast-dividing cells specifically in an effort to stop cancer’s unstoppable spread and eventually eliminate the cancerous cell population. But this treatment also kills normal cells; consequently, it causes serious side effects, such as vomiting, diarrhea, hair loss, bone marrow dysfunction, and lethargy. Currently, the routine treatment for TNBC patients is chemotherapy, but in addition to serious toxicity effects (vomiting, diarrhea, hair loss, bone marrow dysfunction, and lethargy), the malignant cells may also develop resistance to anticancer drugs and evolve into highly drug-resistant cells [8,9]. Hence, targeted therapy, which specifically attacks cancer cells, and immunotherapy have been widely used, but their effects and applications are limited; furthermore, resistance problems occur during long-term treatment.

Recently, the World Health Organization (WHO) announced that more than 2.3 million cases of breast cancer per year, which is the most common disease among women, are reported. The death rate of women due to breast cancer is increasing day by day and become a remarkable cause in almost 95% of countries. In 2020, a study conducted by the International Agency for Research on Cancer reported that nearly 4.4 million women died in 2020 because of cancer, of which 25% of women died due to breast cancer [10,11].

The research studies pointed out that significant contributions In the progression of breast cancer might be implicated by epigenetic controls and non-coding RNAs and impactfully affect the variety and spread of the disease, especially in the context of triple-negative breast cancer (TNBC). It can originate from molecular, cellular, and genetic abnormalities or dysregulation [12,13].

Therefore, this study is conducted to recognize risk factors and predictive models based on the clinical characteristics of the cancer subjects by classifying them into TNBCs (triple-negative breast cancer) and non-TNBCs (one or more positive receptors). Based on this, the health promotion plan for breast cancer patients is to conduct early screening as the primary prevention. This is because early screening can detect potential health problems. If breast cancer is diagnosed through early screening, active cancer treatment, and secondary prevention should be performed by blocking the risk factors identified in studies based on epidemiological characteristics. This may block further disease progression or complications. And it is because you can secure the opportunity to apply various treatment methods.

Accordingly, the specific research goals are as follows: First, the clinical characteristics of TNBCs and non-TNBCs were compared and analyzed. Second, the influencing factors were analyzed through regression and tree models, focusing on statistically significant variables among the clinical characteristics of TNBCs and non-TNBCs.

### The Literature Review

Breast cancer is classified according to gene expression and immunohistochemical staining. Breast cancer is classified into the type A group (estrogen receptor^+^, HER2^−^), the type B group (estrogen receptor^+^, HER2^+^), the HER2 overexpression group (estrogen receptor^−^, HER2^+^), and the estrogen by RNA microarray method. The receptor- and HER2-negative subjects are classified into the basal type group and the normal type group [14,15]. The classification method, according to immunohistochemical staining, evaluates breast cancer by the presence or absence of positive immunohistochemical staining. According to immunohistochemical staining, breast cancer can be divided into TNBCs without estrogen and progesterone receptors, HER2 and non-TNBCs with estrogen and progesterone receptors, and HER2 [4,16].

The gene expression pattern-based classification method is difficult to apply clinically, and currently, a method of distinguishing breast cancer by the presence or nonexistence of hormone receptors and HER2 expression through immunohistochemical staining is used [16]. In gene expression-based breast cancer classification, it is known that the prognosis of the HER2 overexpression group (estrogen receptor^−^, HER2^+^) can improve with targeted therapy, and the basal type group (estrogen receptor^−^, HER2^−^) has many early recurrences and poor prognoses [16]. In the immunohistochemical staining-based classification, when both ER and PR are positive, as opposed to cases where only one receptor is positive (estrogen receptor^+^, progesterone receptor^−^ or estrogen receptor^−^, progesterone receptor^+^), hormone treatment is known to have a good prognosis [5].

The five-year and ten-year survival rates were better when both hormone receptors were positive rather than negative, and survival rates were better when at least one hormone receptor was positive. Even when they were analyzed by stage, the survival rate was better in the hormone receptor-positive cases [17].

In the classification based on immunohistochemical staining, it is known that ER- and PR-positive breast cancer accounts for 75–80%, and HER2-positive breast cancer accounts for 15–20%. TNBC lacking ER, PR, and HER2 expression accounts for 10–15% [7,18]. TNBC is particularly common in those under the age of 40, often called early breast cancer. In addition, because of its aggressive characteristics, it has a rapid rate of cancer progression and a high risk of metastasis and recurrence [19]. In fact, one in three patients with TNBCs experience distant metastasis, where cancer has spread to sites distant from the breast, and overall survival after metastasis averages only from 1 to 1.5 years [19]. Moreover, non-TNBCs are characterized by metastasis to the bone, but TNBCs often metastasize to the brain and lungs, which can have a more fatal effect [19].

ER and PR are intracellular receptors that can be directly measured in tumor tissue [20] and are expressed in about 75–80% of all breast cancer patients [20,21]. Although TNBC does not exactly match the basal type of gene expression, it is sometimes used interchangeably because it shows a similar clinical course [22].

It is known that TNBC patients have a relatively common clinical recurrence, distant metastasis, and poor prognosis compared to non-TNBC patients. In addition, distant metastasis and mortality are high, and a high recurrence is reported within four years after diagnosis [22]. The poor prognosis of TNBC is thought to be related to histopathologic characteristics different from those of non-TNBCs and differences in recurrence and metastasis [4].

Therefore, this research was conducted to recognize the risk factors and analytical models based on the clinical characteristics of breast cancer subjects classified as TNBCs and non-TNBCs.

## 2. Research Method

### 2.1. Research Subjects

The research subjects were 2300 breast cancer patients registered in South Korea in 2016. This study was conducted on 2045 patients who had cancer and underwent all three types of ER, PR, and HER2 tests, and 255 circumstances were excluded because ER, PR, and HER2 were not performed.

Statistics were attained from collaborative stage data from the National Cancer Incidence Database. The American Joint Committee on Cancer (AJCC) surveillance and stage partner organization developed a standardized and integrated staging system, which is called the collaborative stage data collection system (CS) [23]. TNM (tumor, node, and metastasis) staging differs from SEER (surveillance, epidemiology, and end outcomes program) staging, which is used in cancer registration in terms of its goals and intentions [14]. Collaborative Stage Data aim to solve resource waste and inconsistency issues between the two staging systems [23].

Since 1980, the KCCR has served as a countrywide hospital-based program for collecting data from 47 training hospitals across the country [24]. As of 2021, yearly reports of cancer data in Korea from 2008 to 2019 were published [24]. The last follow-up date was 31 December 2018 [24]. These data were approved by the National Statistical Office (Approval No. 117044). The basis for approval is in accordance with Article 14 of the Cancer Control Act and Article 18 of the Statistical Act [25].

### 2.2. Research Tools

The tools of this study set included clinical characteristics, such as age, as independent variables and TNBCs and non-TNBCs as dependent variables.

### 2.3. Clinical Characteristics

Ages included in this analysis were divided into the following groups: ≤39 years; 40–49 years; 50–59 years; 60–69 years; and ≥70 years. Topographic code (T-code), morphologic code (M-code), tumor size, differentiation grade, cancer stage, SEER stage, and neoadjuvant therapy were the clinical parameters considered in this analysis. T-codes were classified from C50.0 to C50.1 (nipple, areola, and central area of the breast), from C50.2 to C50.3 (inner quadrants), from C50.4 to C50.5 (outer quadrants), C50.8 (overlapping lesion), C50.6, and C50.9 (other areas). M-codes were classified as M850–M854 (ductal and lobular neoplasm) and other tissue forms. Tumor size was classified as less than 2 cm and more than 2 cm. The differentiation grade was defined as moderate, poor, and unknown. The four stages of cancer are I, II, III, and IV. Regional, localized, and distant metastases were divided into SEER stages. Neoadjuvant therapy was classified as present, absent, and unknown.

### 2.4. TNBC and Non-TNBCs

The dependent variables included in this analysis, the categories TNBCs and non-TNBCs, are either helpful or harmful for hormone receptors and HER2. Cases that were harmful for estrogen, progesterone, and HER2 were defined as TNBCs, and cases that were positive for any of these three receptors were classified as non-TNBCs. The classification of breast cancer by hormone type is shown in Table 1.

### 2.5. Data Analysis

The clinical characteristics and prognosis of TNBCs and non-TNBCs were compared. All data were evaluated using IBM SPSS 26.0 statistics, Korean edition. The analysis packages were performed in Microsoft Windows 10. An X^2^ test was used to compare TNBC and non-TNBC. Furthermore, the influencing factors of the two groups were analyzed through regression and tree models. First, the numerical results of the influencing factors of TNBCs and non-TNBCs were confirmed by binary logistic regression. Next, the TNBC influencing factors identified in logistic regression were classified and predicted into a decision-making tree using classification CART (regression tree) and CHAID (chi-squared automatic interaction detection) methods. CART, developed by Leo Brineman and Associates in 1984, and CHAID, developed by J. A. Hartigan in 1975, are the most widely used decision-making tree algorithms [26].

CART [27] uses the Gini index as the separation criterion for categorical target variables. The Gini index is one of the measures used to measure the impurity or diversity of each node. It is expressed as

$$G=\sum_{j=1}^{c}P(j)\left(1-P\left(j\right)\right)=1-\sum_{j=1}^{c}P{\left(j\right)}^{2}=1-\sum_{j=1}^{c}{\left(n_{j}/n\right)}^{2}$$

Here, n is the number of observations included in the node. ni is the number of observations in the i-th category of the target variable.

CHAID [28] is an algorithm that completes a multiway split by an X^2^ test (definite target variables) or F-test (constant target variables). This algorithm uses Pearson’s X^2^ test or likelihood-ratio X^2^ test as the separation criterion when the target variable is categorical. Here, the likelihood ratio X^2^ test is used when a target variable is ordinal or grouped continuously. The Pearson’s X^2^ test and likelihood-ratio X^2^ test statistics are expressed as

$$x^{2}=\sum_{i,j}\frac{(f_{ij}-e_{ij}{)}^{2}}{e_{ij}}x^{2}=2\sum_{i,j}f_{i,j}\times {log}_{e}(\frac{f_{ij}}{e_{ij}})$$

Here, f_ij_ is the observation frequency, and e_ij_ is the expected frequency.

A decision-making tree is a knowledge discovery technique used for classification purposes. It is a classified tree, and it is differentiated into branches and leaves from the root node [29]. The decision-making tree model is composed of the structure of “If A, then B. Otherwise, B2”. This means that if A is the case, go to B; otherwise, go to B2 [30].

## 3. Results

### 3.1. Distribution of ER, PR, and HER2

According to Table 2, there were 253 (12.4%) TNBC subjects and 1792 non-TNBC subjects, accounting for 87.6% of the total. Among non-TNBCs, ER, PR helpful, and HER2 harmful were the highest in 1047 (51.2%) patients. Triple-positive breast cancer (TPBC) was 261 (12.8%), and hormone receptor-negative and HER2-positive were 205 (10.0%).

### 3.2. Cross-Analysis between T-Code and M-Code

Ductal and lobular tissue breast cancer occurred most frequently in the outer quadrant (C50.4–C50.5) in 833 patients (43.1%). In other tissue breast cancers, overlapping lesions (C50.8) were 390 (20.2%), and the inner quadrant (C50.2–C50.3) was 375 (19.4%). A cross-analysis between T-code and M-code is shown in Table 3.

### 3.3. Clinical Characteristics Comparison of TNBCs and Non-TNBCs

According to Table 4, TNBC was found in 38 patients (19.5%) under the age of 39, followed by 33 patients (17.3%) over the age of 70. Non-TNBCs were the most frequent in the 40–49-year group, with 603 patients (90.7%), followed by 581 patients (88.3%) in the 50–59 year group. There was a statistically significant age change among the two groups (*p* = 0.001).

For TNBC, the T-code of C50.4–C50.5 had the highest frequency in 125 cases (14.4%), followed by C50.8 in 52 cases (12.6%). For non-TNBCs, C50.6 and C50.9 had the highest frequency in 222 cases (91.0%), followed by C50.0–C50.1 in 108 cases (90.8%). There was no statistically significant change in the T-codes among the two groups (*p* = 0.104).

Likewise, the M-code of TNBC showed the highest frequency in 20 cases (18.0%) in tumors of other tissue forms. There were 233 cases (12.0%) of ductal and lobular tumors (M850–M854). For non-TNBCs, 1701 (88.0%) ductal and lobular tumors (M850–M854) were more frequent than other forms of tissue tumors 91 (82.0%). Among the two groups, there was no statistically significant change in M-code (*p* = 0.074).

Regarding tumor size, TNBC was more than 2 cm in 159 cases (16.0%) and less than 2 cm in 94 cases (9.0%). Non-TNBCs were less than 2 cm in 956 cases (91.0%) and more than 2 cm in 836 cases (84.0%). There was a statistically significant change in tumor size among the two groups (*p* = 0.001).

As for the differentiation grade in the TNBC, poorly differentiated had the highest incidence at 45 cases (24.7%), followed by an unknown differentiated with 189 cases (12.3%). Non-TNBCs were well-differentiated in 83 cases (98.8%) and moderately differentiated in 225 cases (92.6%). The distinction between the two groups’ differentiation grades was statistically important (*p* = 0.001).

Among the TNBC cases, stage IV was the most common in 15 cases (21.7%), followed by stage II in 127 cases (15.7%). In non-TNBC cases, stage I was the most common in 839 cases (90.8%), followed by stage II in 212 cases (89.1%). Among the two groups, there was a statistically significant change in the cancer stage (*p* = 0.001).

For the SEER stage, TNBC had 17 cases (21.0%) with distant metastasis (code 7) and 156 cases (12.3%) with localized metastasis (code 1). On the other hand, non-TNBCs had 614 cases (88.5%) with regional metastasis (code 2–4), which was the most frequent, followed by 1114 cases (87.7%) with localized metastasis (code 1). A statistically significant change in the SEER stage existed among the two groups (*p* = 0.049).

Finally, neoadjuvant therapy for TNBC was received in 26 cases (24.1%), whereas 204 cases (11.3%) did not receive treatment. For non-TNBCs, 1609 cases (88.7%) did not receive treatment, and 82 patients (75.9%) received neoadjuvant therapy. It was statistically significant (*p* 0.001) that the two groups differed in their use of neoadjuvant therapy.

### 3.4. Clinical Characteristics as Factors Associated with TNBC

A study using logistic regression was conducted to determine the clinical traits that were factors influencing the differences between the TNBC and non-TNBC groups (Table 5). Age, T-code, differentiation grade, SEER stage, and neoadjuvant therapy were identified as factors associated with TNBC. M-code is also associated with the TNBC. It might be possible that the association of the T-code is more prominent than that of the M-code.

Standard in the age of 39 years or younger, TNBC was 2.12 times (*p* = 0.001) more prevalent in those aged 40–49 and 1.78 times (*p* = 0.012) more likely to occur in people aged 50–59. Standard in the ductal and lobular tumors (M850-M854), TNBC was 0.55 times (*p* = 0.025) less likely in other forms of tissue. Standard on the well-differentiation scale, TNBC is 0.04 times (*p* = 0.002) less likely to be poorly differentiated. Standard in the SEER code localized, TNBC was 1.61 times (*p* = 0.020) more likely to be regional in SEER. Standard in the neoadjuvant therapy, TNBC was 2.66 times (*p* < 0.001) more likely to be a non-treated group. Incidentally, T-code, tumor size, and cancer stage were not statistically significant factors.

### 3.5. Prediction Model According to the Presence or Absence of TNBC

**Figure 1:**
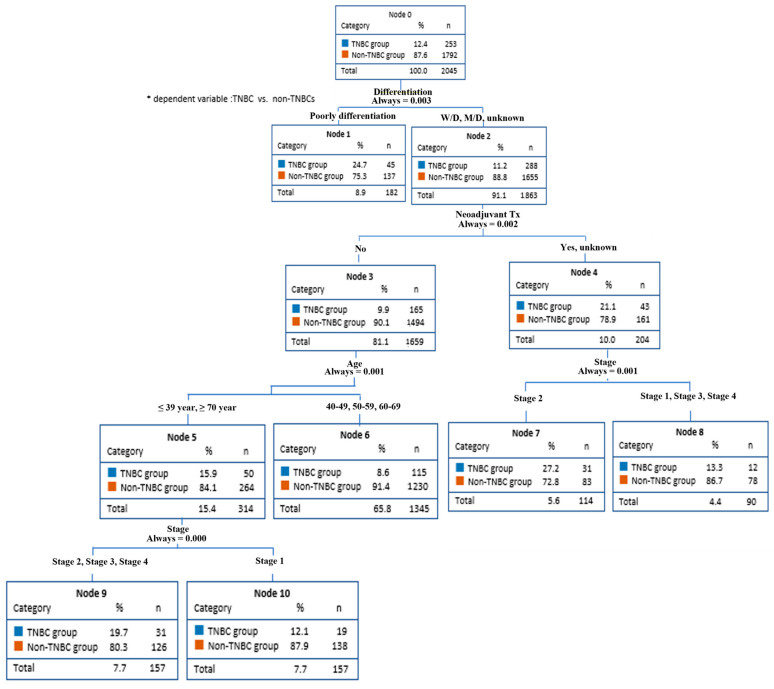
Breast cancer prediction model (CART).

The decision-making tree methods (CART and CHAID), also known as decision-making trees, have been used to predict factors in TNBC, showing the nodes of the decision-making tree, from the main node to the fatal node, and the separation criteria. Through CART method analysis, TNBC predictors were found for differentiation grade, neoadjuvant therapy, age, and cancer stage (Figure 1). On the other hand, the CHAID method analysis found TNBC predictors for differentiation grade, neoadjuvant therapy, and age.

This model predicted that the non-TNBCs had an 88.8% probability of all differentiation grades (node 2) except for poorly differentiated. The differentiation grade affected neoadjuvant therapy. The non-TNBC group had a 90.1% chance of not receiving neoadjuvant therapy (node 3). In addition, because neoadjuvant therapy affects age, patients aged <39 years and >70 years (node 5) had an 84.1% chance of being included in the non-TNBCs group. Finally, age influences the stage of cancer. Stages II, III, and IV (node 9) had an 80.3% probability of being in the non-TNBC group.

On the other hand, the non-TNBC group had a 78.9% probability of undergoing neoadjuvant therapy or unknown (node 4). Since neoadjuvant therapy affected the stage, there was a 72.8% probability of stage II being in the non-TNBC group (node 2).

**Figure 2:**
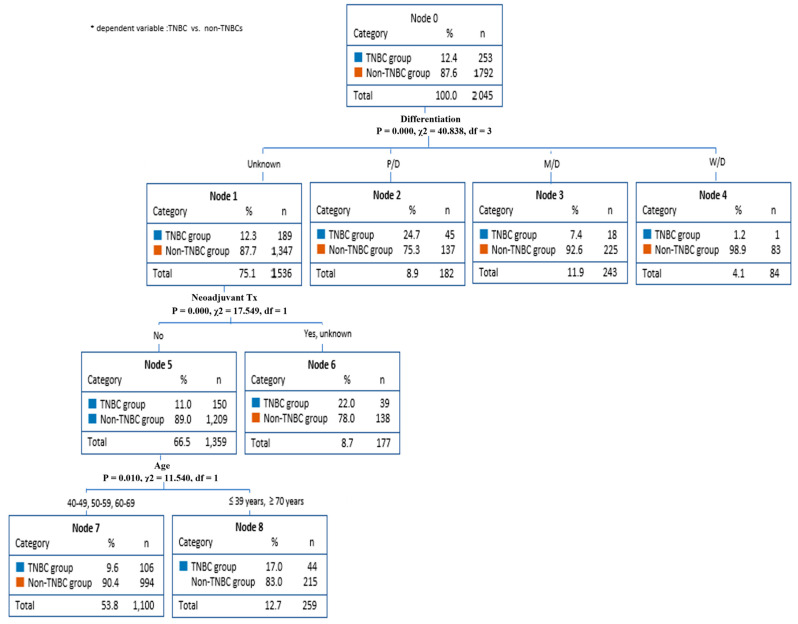
Breast cancer prediction model (CHAID).

Figure 2 shows that the decision-making tree method by CHAID was used to predict factors in TNBC. This analysis found TNBC predictors for differentiation grade, neoadjuvant therapy, and age. Specifically, this model predicted that the non-TNBCs had an 87.7% probability of unknown differentiation (node 1). Differentiation grade influences neoadjuvant therapy. The non-TNBCs group had an 89.0% chance of not receiving neoadjuvant therapy (node 5). In addition, because neoadjuvant therapy affects age, patients aged 40–60 years (node7) had a 90.4% chance of being included in the non-TNBCs group.

Table 6 is a risk chart for evaluating the breast cancer prediction model according to the presence or absence of TNBC. A risk estimate indicates the risk of being misclassified or predicted, and the smaller this value, the more successful the model building is [31].

The possibility estimation of this model was 1.954 in the logistic regression study; the standard error (SE of risk estimate) was 0.067, and the classification correctness was 87.8%. The decision-making tree classification accuracy was 87.6%; the standard error was 0.007, and the risk estimate was 0.124.

## 4. Discussion

The TNBC is multifaceted, and breast cancer subtypes lack the expression of ER, PR, and HER2, thereby causing difficulty in therapeutic targeting. It generally has a high malignancy rate and a poorer prognosis [32]. TNBC accounts for 12.4% of breast cancer cases, and the relative incidence is higher in individuals younger than 39 and older than 70. The following variables were also relatively higher in TNBCs than in non-TNBCs. The tumor size was more than 2 cm; the stage was IV, and the SEER was distant metastasis (code 7).

Among patients with cancer, the percentage of TNBC patients was 24.6–31.6% for African American women, 10.8–23% for Caucasian women, and 15% for Japanese women, showing slightly different patterns, depending on race [33,34,35,36,37]. In the present study, 12.4% of patients were determined to be TNBC, and this value was lower than the overseas incidence rate of TNBC. ER (+)/PR (+) and HER2 (−) showed the largest distribution with 51.2%. The anatomical sites of tumor development most frequently occurred in the outer quadrant (C50.4–C50.5) and in ductal and lobular areas (M850–M854). The breast is an anatomically tubular region with a high incidence in the axillary proximal lateral quadrant, which is rich in lymph nodes.

As for the frequency of occurrence by age, TNBC was more common in those aged under 39 and over 70, and non-TNBCs were more common in those in their 40s and 50s. Similar to these study results, previous findings showed that the occurrence of TNBC is high among individuals aged <35 years [38]. The occurrence of TNBC is high in the age group with low incidence, not in the 40–50 age group, where breast cancer is common. As for the size of the tumor in this study, 62.8% of TNBC larger than 2 cm were reported. Dent et al. [22] reported that approximately 70% of the TNBC patient group had tumors larger than 2 cm, and Ahn et al. [38] reported 55.5%. TNBC has a higher proportion of tumors of >2 cm than those of <2 cm. This finding suggests that TNBC tumors are more likely to develop when tumors are larger than 2 cm in size. Histological differentiation is worse in TNBC [22,39,40], and histological and nuclear differentiation in TNBC is worse than that in non-TNBCs [22,40]. In the present study, TNBC showed a high rate of poor differentiation.

The TNBC group has a relatively poorer prognosis than the non-TNBC group, likely because of the lack of a known therapeutic target such as hormone receptor or HER2 expression, but no specific treatment method, other than nonspecific chemotherapy, is available [6]. The prognosis was poor as the ratio of stage IV to distant metastasis was high even in the stage of cancer or SEER of cancer. Similar to previous findings [38,41], the present study result showed that the TNBC group received more neoadjuvant therapy than the non-TNBC group.

As a result of the CART and CHAID methods of decision-making trees for predicting risk factors of TNBC, differentiation degree, neoadjuvant therapy, and age were predicted to be common factors, and the high prediction rate was 87.6%. In this study, breast cancer subjects were retrospectively analyzed. The prospective approach of TNBC, which targets women under 40 and over 70 with a high incidence of TNBC, requires regular self-examinations, imaging education through multiple channels, and national cancer screening.

Therefore, preventive interventions are very necessary. In addition, if cancer has occurred, the differentiation status must be evaluated, and a treatment approach that prioritizes neoadjuvant therapy is necessary. On the other hand, TNBC cannot be treated with hormone therapy. Since targeted therapeutics such as Herceptin^®^ (trastuzumab) cannot be used, only chemotherapy is used for neoadjuvant therapy [37]. The main anticancer agents used as neoadjuvant therapy before surgery and postoperative adjuvant therapy are anthracycline and taxane systems [38].

This retrospective study was conducted to analyze the incidence of TNBC and non-TNBC breast cancer in Korean women and to examine its clinical characteristics and risk factors. In future studies, a prospective approach based on the risk factors identified in this study or a cohort study through continuous observation should be performed.

## 5. Conclusions

This research was conducted to identify the risk factors and predictive models based on the clinical characteristics of breast cancer subjects classified as TNBCs and non-TNBCs using Korean cancer statistics. The main research results and conclusions are as follows:

The occurrence of TNBC is the most common in patients aged ≤39, followed by those aged ≥70. Tumors larger than 2 cm had more frequent occurrence than tumors smaller than 2 cm. Poor differentiation was the highest. Additionally, stage IV was the most common, and SEER was in distant metastasis (code 7). The performance of neoadjuvant therapy was higher than that of non-treatment.

Logistic regression analysis revealed that age, M-code, differentiation grade, SEER stage, and neoadjuvant therapy were factors associated with TNBC. Conversely, decision-making tree analysis showed that differentiation grade, neoadjuvant therapy, and age were factors associated with TNBC. Both predictive models identified age, differentiation grade, and neoadjuvant therapy as influencing factors.

Therefore, the therapeutic approach for patients with TNBC should consider differentiation grade, neoadjuvant therapy, age, and staging. In addition, patients with TNBC should be carefully observed and actively treated because of their poor differentiation, high staging, and the chance of distant metastases is considerable. To promote the health of breast cancer patients, it is urgent to apply an intensive early health check-up program for those in their 40s and 50s with a high prevalence of TNBC. This allows for the early discovery of breast cancer and screening for TNBC-positive patients. The establishment of a policy system is necessary for the success of this process.

Since this study applied clinical characteristics and risk factors of breast cancer patients to Koreans, there is a limitation in generalizing it globally. However, it is meaningful to study pathological characteristics and risk factors for health promotion in Korean breast cancer patients using a retrospective method. Additional variables that may lead to breast cancer in the future, mortality, and their relationship to other factors should be analyzed in detail. In addition, it is necessary to understand the characteristics of each country, race, and continent through comparative studies between Korea and other countries. Therefore, these studies are extremely pertinent for research purposes and can offer guidance for analyzing the statistics of patients with breast cancer in diverse communities from various regions.

## Figures and Tables

**Table 1:** Classification of breast cancer by hormone type.

ER, PR	HER2	Classification of Breast Cancer	Note
+	−	ER/PR-positive	Non-TNBCs
−	+	HER2-positive	
+	+	ER/PR- and HER2-positive	
−	−	Triple-negative	TNBCs

**Table 2:** Distribution of ER, PR, and HER2.

Classification	*n*	%	Cumulative (%)	X^2^ (p)
Triple-negative	253	12.4	12.4	2045.000 (0.000)
Triple-positive	261	12.8	25.2	
ER (+) PR (+) HER2 (−)	1047	51.2	76.4	
ER (−) PR (−) HER2 (+)	205	10.0	86.4	
ER (+) PR (−) HER2 (−)	166	8.1	94.5	
ER (+) PR (−) HER2 (+)	95	4.6	99.1	
ER (−) PR (+) HER2 (+)	10	0.5	99.6	
ER (−) PR (+) HER2 (−)	8	0.4	100.0	
Total	2045	100.0		

**Table 3:** Cross-analysis of T-code and M-code in breast cancer.

Classification	T-Code *n* (%)					Total *n* (%)	X^2^ (*p*)
	C50.0–C50.1	C50.2–C50.3	C50.4–C50.5	C50.8	C50.6 –C50.9		
M850–M854	113 (5.8)	375 (19.4)	833 (43.1)	390 (20.2)	223 (11.5)	1934 (100.0)	26.550 (0.047)
M844–M849	3 (6.5)	10 (21.7)	17 (37.0)	10 (21.7)	6 (13.0)	46 (100.0)	
M814–M838	1 (3.3)	3 (10.0)	14 (46.7)	6 (20.0)	6 (20.0)	30 (100.0)	
M856–M857	-	6 (46.2)	4 (30.8)	2 (15.4)	1 (7.7)	13 (100.0)	
M839–842, M800	2 (9.1)	4 (18.2)	3 (13.6)	5 (22.7)	8 (36.4)	22 (100.0)	
Total *n* (%)	119 (5.8)	398 (19.5)	871 (42.6)	413 (20.2)	244 (11.9)	2045 (100.0)	

Note (1) C50.0–C50.1: nipple, areola, central portion; C50.2–C50.3: inner quadrant of breast; C50.4–C50.5: outer quadrant of breast; C50.8: overlapping lesion of breast; C50.6, C50.9: other; Note (2) M850–M854: ductal and lobular neoplasm; M844–M849: cystic, mucinous, and serous neoplasm; M814–M838: adenocarcinoma; M856–M857: complex epithelial neoplasm; M839–M842, M800: other.

**Table 4:** Comparison of clinic characteristics between TNBCs and non-TNBCs.

Characteristics		TNBC *n* = 253 (%)	Non-TNBCs *n* = 1792 (%)	Total *n* = 2045 (%)	X^2^ (*p*)
Age	≤39 years	38 (19.5)	157 (80.5)	195 (100.0)	19.376 (0.001)
	40–49 years	62 (9.3)	603 (90.7)	665 (100.0)	
	50–59 years	77 (11.7)	581 (88.3)	658 (100.0)	
	60–69 years	43 (12.8)	293 (87.2)	336 (100.0)	
	≥70 years	33 (17.3)	158 (82.7)	191 (100.0)	
T-code	C50.0–C50.1	11 (9.2)	108 (90.8)	1199 (100.0)	7.677 (0.104)
	C50.2–C50.3	43 (10.8)	355 (89.2)	398 (100.0)	
	C50.4–C50.5	125 (14.4)	746 (85.6)	871 (100.0)	
	C50.8	52 (12.6)	361 (87.4)	413 (100.0)	
	Other	22 (9.0)	222 (91.0)	244 (100.0)	
M-code	M850-M854	233 (12.0)	1701 (88.0)	1934 (100.0)	3.452 (0.074)
	Other	20 (18.0)	91 (82.0)	111 (100.0)	
Tumor size	<2 cm	94 (9.0)	956 (91.0)	1050 (100.0)	23.273 (0.000)
	≥2 cm	159 (16.0)	836 (84.0)	995 (100.0)	
Differentiation	Well	1 (1.2)	83 (98.8)	84 (100.0)	40.838 (0.000)
	Moderate	18 (7.4)	225 (92.6)	243 (100.0)	
	Poor	45 (24.7)	137 (75.3)	182 (100.0)	
	Unknown	189 (12.3)	1347 (87.7)	1536 (100.0)	
Stage	Stage I	85 (9.2)	839 (90.8)	924 (100.0)	23.908 (0.000)
	Stage II	127 (15.7)	681 (84.3)	808 (100.0)	
	Stage III	26 (10.9)	212 (89.1)	238 (100.0)	
	Stage IV	15 (21.7)	54 (78.3)	69 (100.0)	
SEER stage	Localized (code 1)	156 (12.3)	1114 (87.7)	1270 (100.0)	6.012 (0.049)
	Regional (code 2~4)	80 (11.5)	614 (88.5)	694 (100.0)	
	Distant (code 7)	17 (21.0)	64 (79.0)	81 (100.0)	
Neoadj. Tx	Yes	26 (24.1)	82 (75.9)	108 (100.0)	20.103 (0.000)
	No	204 (11.3)	1609 (88.7)	1813 (100.0)	
	Unknown	23 (18.5)	101 (81.5)	124 (100.0)	

**Table 5:** Influencing factors on clinical characteristics according to TNBC.

Classification		Exp (B)	95% CI	*p*
Age	≤39 years	1.000		
	40–49 years	2.123	1.342–3.359	0.001
	50–59 years	1.775	1.136–2.775	0.012
	60–69 years	1.537	0.929–2.541	0.094
	≥70 years	1.002	0.580–1.729	0.996
T-code	C50.0–C50.1	1.000		
	C50.2–C50.3	0.745	0.361–1.537	0.425
	C50.4–C50.5	0.530	0.270–1.042	0.066
	C50.8	0.642	0.315–1.307	0.222
	Other	0.932	0.423–2.052	0.861
M-code	M850-M854	1.000		
	Other	0.547	0.323–0.928	0.025
Tumor size	<2 cm	1.000		
	≥2 cm	0.695	0.420–1.149	0.156
Differentiation	Well	1.000		
	Moderate	0.154	0.020–1.178	0.072
	Poor	0.040	0.005–0.302	0.002
	Unknown	0.092	0.013–0.668	0.018
Stage	Stage I	1.000		
	Stage II	0.721	0.409–1.272	0.258
	Stage III	1.067	0.469–2.425	0.877
	Stage IV	0.452	0.070–2.910	0.403
SEER stage	Localized (code 1)	1.000		0.064
	Regional (code 2~4)	1.610	1.080–2.402	0.020
	Distant (code 7)	1.401	0.262–7.489	0.694
Neoadj. Tx	Yes	1.000		0.000
	No	2.656	1.577–4.472	0.000
	Unknown	1.535	0.792–2.978	0.205

**Table 6:** The prediction rate of regression and decision-making tree models.

Observed			Prediction Category				Risk Estimate	SE of Risk Estimate
			TNBC	Non-TNBCs	Total	Predicted Ratio		
Logistic regression	Actual category	TNBC	13	240	253	5.1	1.954	0.067
		Non-TNBCs	9	1783	1792	99.5		
Total			22	2023	2045	87.8		
Decision-making tree	Actual category	TNBC	0	253	253	0.0	0.124	0.007
		Non-TNBCs	0	1792	1792	100.0		
Total			0	2045	2045	87.6		

## Data Availability

Data are contained within the article.
